# First Clinical Research Informatics (CRI) Solutions Day: advanced IT support from EU projects for clinical trials

**DOI:** 10.1186/2043-9113-5-S1-A1

**Published:** 2015-05-22

**Authors:** Wolfgang Kuchinke, Töresin Karakoyun, Simon Gengler, Christian Ohmann

**Affiliations:** 1 Coordination Centre for Clinical Trials, Düsseldorf University Hospital, Düsseldorf, Germany; 2 European Clinical Research Infrastructures Network (ECRIN), KKS, Düsseldorf, Germany

## Necessity for a novel form of interactive conference

Clinical trials are the foundation for the advancement of medical research, but they are also complex, time consuming and expensive. To ensure scientific validity, clinical trials must record large amounts of data on health and treatment of a carefully selected group of patients or trial participants. That is why a robust and highly flexible IT infrastructure that permits rapid reconfiguration to support different trials while maintaining consistent data models is needed to ensure that clinical data can be shared and used for analysis. Recently, EU projects, like TRANSFoRm, p-medicine, EHR4CR and BioMedBridges, are creating advanced clinical research information systems consisting of sets of tools that assist in the preparation and conduct of clinical trials and the re-use of care data for research purposes. These tools are also able to assemble data from heterogeneous sources to answer complex research questions, including functions for data mining and support of research process workflows to meet the needs of translational and personalized medical research [1].

Because the translation of biomedical discoveries into clinical applications is an aim of the EU Commission, clinical research support is expected to be a major component of EU funded projects [2]. In this context, the EU Framework Program 7 (FP7, http://ec.europa.eu/research/fp7/index_en.cfm) has promoted translational research, the so-called ‘from bench-to-bedside’ approach, that is expected to have practical benefits and improvements of the quality of life for patients. In this context, it is planned that many of the tools developed in ESFRI (European Strategy Forum on Research Infrastructures) and IMI projects will be used by ECRIN, the European Clinical Research Infrastructures Network [3] to advance clinical trials. To provide information on these new research tools and to evaluate their usability for the clinical research community, the CRI Solutions Day was organised by ECRIN together with other EU-funded projects (TRANSFoRm, EHR4CR, p-medicine, BioMedBridges and ECRIN-IA) providing interactive “hands-on” sessions to allow visitors to see these tools in action. The necessity for such a solutions day was based on the experience of ECRIN that the conventional, class room style presentation of research tools is not sufficient to demonstrate comparability and usability to many researchers. Thus, presenters from academia, research infrastructures and EU projects presented their developments with interactive sessions embedded in a framework of presentations and joint discussions. The CRI solutions day took place at Heinrich-Heine University Düsseldorf, in Düsseldorf, Germany, 26-27 May 2014.

## A new landscape for modern clinical research

**Figure 1:**
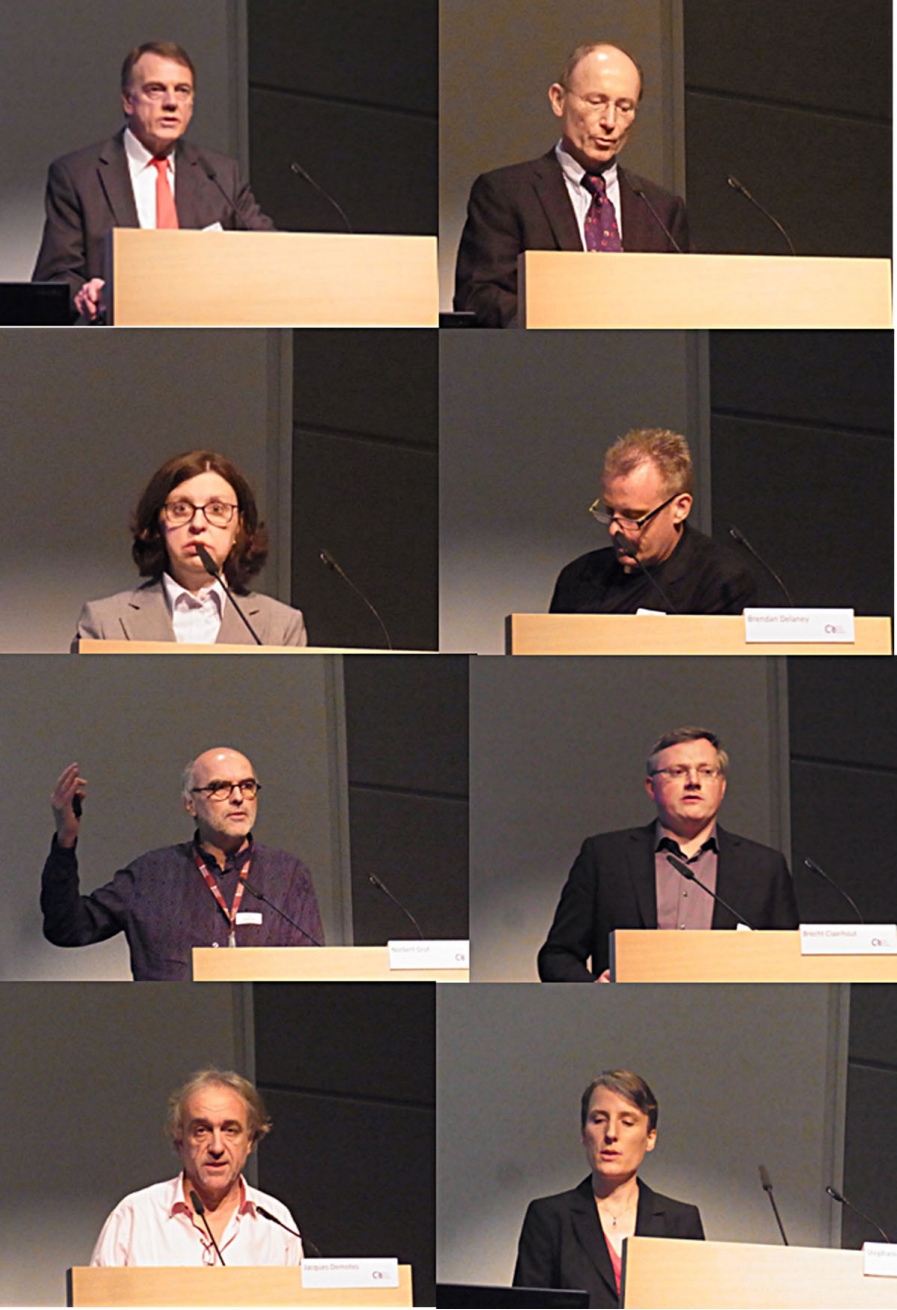
Key notes speakers and EU project heads (from top to bottom: Christian Ohmann, Michael Piper, Ann Martin, Brendan Delaney, Norbert Graf, Brecht Claerhout, Jacques Demotes, Stephanie Suhr).

After the welcoming speech by the Rector of Heinrich-Heine University **Michael Piper (Heinrich-Heine University Düsseldorf)**, **Christian Ohmann (KKS and ECRIN)** opened the conference with an overview on the current developments in clinical trials and the influence that translational medicine and data-driven science may have on clinical trials progress (Figure 1). Because still only very few innovations have managed to get into clinical routine use, the need for change in drug development is evident. It was proposed that data driven research will be able to discover structures in big data and lead to predictions avoiding the detour through hypothesis-driven research and clinical trials. One should ask, if it is possible to apply a unified modeling approach to health care, and improve the effectiveness of health care with data-driven medicine using for example genome-wide research data. But data-intensive science may rather complement conventional methods and thus be used to increase potential benefits following clinical trials to demonstrate efficacy and efficiency. For example, it may be possible to use big data to identify subgroups of patients that will profit from a treatment; or test oncological drugs to receive a better therapeutic prediction. Whatever the case, the clinical trials landscape will change; EHR data will be used for feasibility and patient recruitment. Increasingly predictive biomarkers will be analysed to determine outcome, increase power and to stratify patients in clinical trials.

**Ann Martin (IMI)** explained the Innovative Medicines Initiative (IMI). IMI is Europe's largest public-private initiative of collaborative research projects aiming to speed up the development of better and safer medicines. About 2 billion Euros are being spent with 50% of the sum paid by the EU and 50% by the pharma industry. It covers until to date 11 calls for proposals resulting in 59 collaborative projects. About 130 Mio Euro is being spent only for improved knowledge management, covering integrated biomedical data platforms and tools for data management and big data (e.g. eTOX, OpenPHACTS, EMIF, eTRIKS). Two important aspects of all EU projects are aspects of descriptive metadata and data quality / data governance. Interoperability must be established by the implementation of standards. IMI II has begun with 3.45 billion Euro and employing H2020 rules, with the first call starting about July 2014. Because IMI projects are focusing on -omics (research including genomics, proteomics or metabolomics), genomics and patient data, the platforms and tools discussed in this solutions day are of importance for IMI projects, too.

## Session 1: EU funded projects with participation of ECRIN

**Wolfgang Kuchinke (Heinrich-Heine University, Düsseldorf)** described the role ECRIN plays in the EU projects TRANSFoRm, p-medicine, BioMedBridges and EHR4CR and how ECRIN plans to employ tools developed in these projects. ECRIN participated in developing information models, data models and ontologies, promoted the use of standards and created legal and ethical frameworks for these projects. Most of the developed tools will be provided as services and these services have to be integrated into the ECRIN infrastructure of clinical trial support. This integration must proceed on three levels: technical level, process level and business level. For ECRIN, the integration of data stored in hospital information systems (HIS) and EHR for research purposes will be a special challenge, requiring the collaboration of local hospitals, EHR and HIS vendors, patients, investigators, ECRIN EU Correspondents and clinical trials units (CTU) staff.

**Brendan Delaney (King’s College, London)** explained the importance of the concept of the Learning Health Care System (LHS) for the TRANSFoRm project [4]. Enabling the use of primary care data for research, the final aim of TRANSFoRm is the improvement of patient health and safety. TRANSFoRm consists of three parts: first, support of epidemiological genotype-phenotype studies; second, support for randomized clinical trials (RCT), and third, decision support for the physician. Pilot studies with the tools developed in TRANSFoRm will begin soon. EHR data sources will be analysed using the Query Workbench. The randomized clinical trial will employ an eCRF integrated in an EHR that provides a trigger mechanism for indicating to the physician eligible patients. To bridge different clinical concepts between the usage of clinical data in care and research, ontologies were implemented in the tools (CDIM). In addition, a full data provenance trail is ensured, and standards, terminologies and archetypes are combined. To illustrate the recent developments of IT in clinical research; Brendan Delaney used the picture of the evolution of the human brain. The legacy systems together with associated terminologies, like HL7, CDISC ODM represent the reptilian brain; the interactive and semantic aspects of IT solutions, like models and archetypes, are represented by the limbic brain and the medical reasoning based on ontologies is represented by the most evolved form, the neocortex.

**Norbert Graf (USAAR)** presented results and challenges of the p-medicine project [5], a clinically driven project about personalized medicine in oncology with 19 partners. The p-medicine platform serves knowledge discovery, including genetic and phenotype data. It allows the assessment of analytical results in context to ensure that patients obtain an individualized therapy. Thus patient empowerment must play an important part in the project. Tools, models, means to ensure re-use of data, and data warehouses are enclosed by a security framework, consisting of data anonymisation, contracts with users and consents given by data donors. Clinicians will be the users of the tools, but p-medicine will also ensure that more patients are enrolled in personalized medicine trials.

**Brecht Claerhout (Custodix)** presented EHR4CR [6], an IMI project that will enable the re-use of hospital data for optimising clinical trials conduct. The need for the project results in the fact that drug innovation has become too slow and too expensive (about 1.2 billion Euro is needed to develop a drug from a chemical entity). Most important, 50% of delays in clinical trials come from inefficient patient recruitment, because clinical trials often fail to enrol enough patients. EHR4CR has four use cases for EHR data re-use: optimizing protocol feasibility, faster patient recruitment, improvement of study conduct by pre-filling eCRFs and finally the capture of adverse events (AE). The services developed will access real clinical trials information by running queries at different clinical sites that compare if criteria are met. This procedure will be able to speed up recruitment by giving physicians and investigators means to search for eligible patients. The EHR4CR project is characterized by an open architecture, a service platform, use of standards and is open to different semantic layers. It has now reached the testing phase of its pilots.

**Jacques Demotes (ECRIN)** presented ECRIN-IA and concentrated on the project’s data management aspects. ECRIN has become an ERIC (European Research Infrastructure Consortium), a legal structure with now 5 countries involved, and 4 other countries that will join soon. ECRIN-IA is about the structuring of user communities for nutritional research, rare diseases and medical devices. Data management is part of ECRIN-IA and started in the previous project with a large survey of all ECRIN centres [7]. This survey showed that data management solutions for clinical trials are very heterogeneous and differences in quality standards exist at data centres. What ECRIN needs is a system easy to use in all ECRIN data centres that is cost effective to implement and extensible. Addressing the need for quality standards in clinical data management, a certification program for ECRIN data centres was developed covering GCP compliance and ECRIN requirements. A suitable data management solution (VISTA) is being built by EORTC according to ECRIN specifications. VISTA will be available at low cost for academic institutions and for ECRIN. In addition, ECRIN plans to develop a clinical trials outcome database that will store core data sets of outcome measures of ECRIN trials to enable further meta-analysis after the end of the trials.

**Stephanie Suhr (BioMedBridges)** presented BioMedBridges, a large EU project in which 21 partners represent 10 new European research infrastructures covering research domains from bioinformatics and biobanking to translational research. Recently, emerging infrastructures for systems biology and microbiology have also joined. The aim of BioMedBridges is to integrate existing islands of data sources that harbour clinical data, biological data, and images in different research infrastructures by the means of data bridges and services. With the help of REST services and a data security framework, data previously isolated will become discoverable for researchers. Data integration will take place on the technical level (standards) and on the process level by REST services and by semantic web techniques. Three tools developed by BioMedBridges were presented at the workshop, CTIM, MOLGENIS and XNAT. In addition, LAT was developed, a tool that precedes the data discovery process and supports researchers in identifying legal and ethical risks associated with the process of data access or data sharing.

The following joint discussion focused on the provision and the sustainability of the multitude of tools developed in EU projects. Because these tools are public developments, they should be freely shared over the web (Ann Martin). In this context all speakers confirmed the future public availability of the tools developed. Stephanie Suhr affirmed that all deliverables and tools developed in BMB will be public. Though, ECRIN deliverables are public, VISTA will not be open source and usage will be accompanied by a non-profit fee. For the p-medicine project, too, all tools will be Open Source (Norbert Graf). All deliverables of TRANSFoRm are publicly available; and TRANSFoRm tools will be provided as Open Source for free usage with accompanying documentation. EHR4CR too will make deliverables publicly available.

A discussion followed about the role that a certification procedure can play in infrastructures and for tool employment. A certification of tools could ensure the GCP compliance of usage or the observance of regulations, like the ones for data protection. ECRIN has developed a certification procedure with an independent certification board for clinical data centres. During the phase of certification development the German Accreditation Agency advised ECRIN not to start with establishing an accreditation, but to create a certification process (Christian Ohmann). Norbert Graf explained that p-medicine has launched a special legal entity to ensure its sustainability: STaRC. All tools in p-medicine must be validated for GCP compliance and usability by clinicians. But an important aspect is that the validation and certification of tools that will be used in clinical trials is out of scope of EU projects. In addition, lawyers are needed to set up contracts between providers, data owners and users. For BioMedBridges, Stephanie Suhr explained, sustainability is built already into the project by the participating research infrastructures which are themselves sustainable entities.

Conference participants noticed similarities in tool development. For example, TRANSFoRm and EHR4CR have developed tools/services with several aspects in common, like the search for patient’s inclusion and exclusion criteria. It was asked, if both projects will not develop tools or deliverables together. But the requirements underlying these projects are quite different. Brendan Delaney explained that for TRANSFoRm the inclusion / exclusion criteria model is based specifically on PCROM and BRIDG, and both models determine how the query workbench can operate and how the queries are created. Design of queries is based on LexEVS terminology [8], and is linked over data node connectors with ontologies. TRANSFoRm is a distributed infrastructure and allows that data stays where it is. In contrast, according to Brecht Claerhout, the EHR4CR project starts from a clinical model, ETL to the data warehouse. All queries are built upon a specific information model (blue model and ECLECTIC) and the concepts of the information model are again mapped to queries.

## Break out session 1

**Figure 2:**
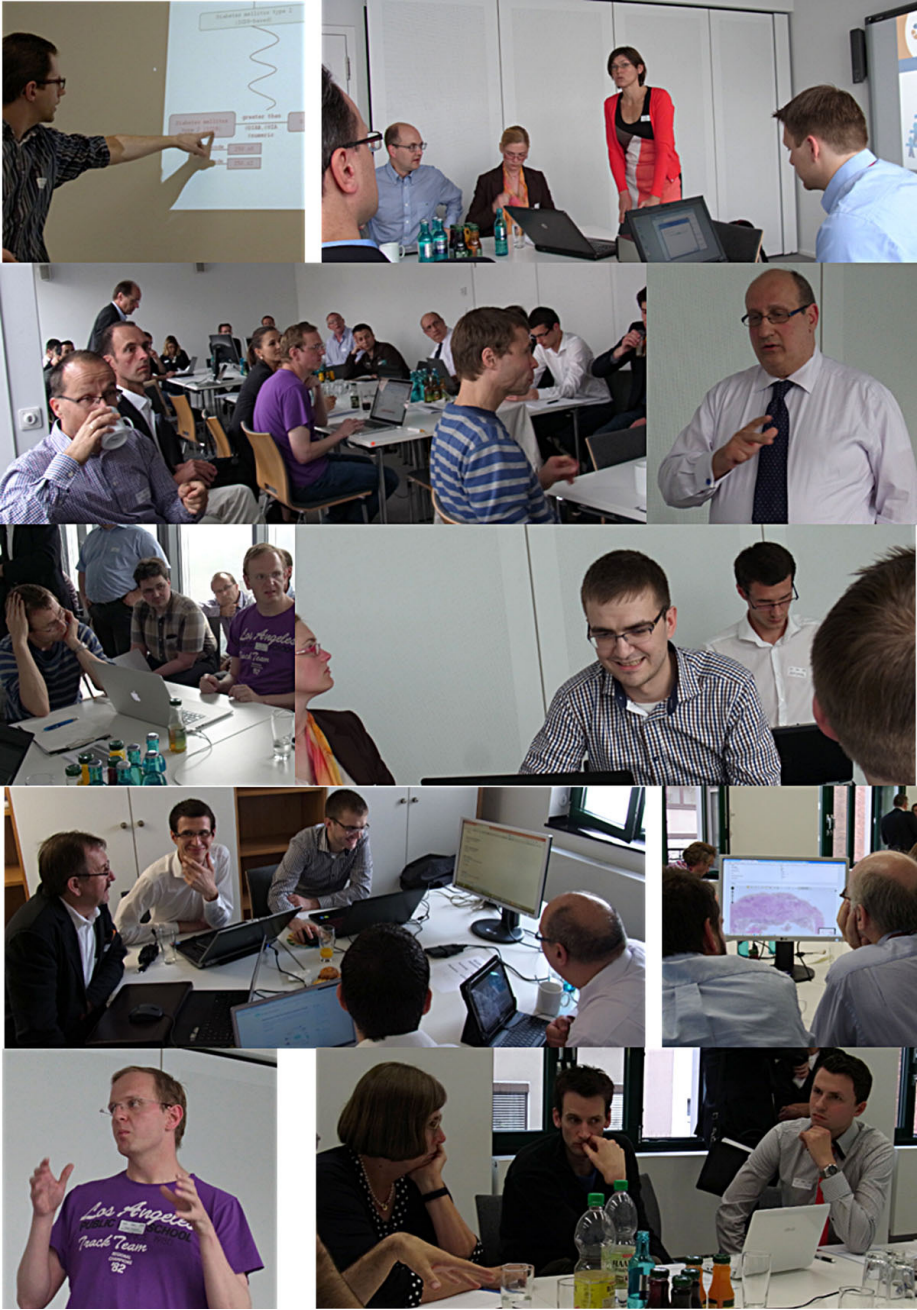
Impressions from the break-out and software demonstration sessions.

In this breakout session (Figure 2), tools for clinical data management (OpenClinica, ObTiMA, VISTA), tools for bridging experimental and clinical research data (tranSMART, MOLGENIS, i2b2), and tools to integrate EHR data for clinical research (Feasibility Service, Patient Screening Tool, Recruitment and Feasibility Tools) were presented and discussed.

## Break out session 2

In the second breakout session (Figure 2), additional tools for clinical data management (Functional eCRF, mobile eHealth Solution), imaging tools for clinical research (XNAT imaging pipeline, DoctorEye), and tools for biobanking in clinical research (p-BioSPRE, Biobanking Catalogue, BBMRI Catalogue) were presented and discussed.

## Panel discussion: what to do with all these tools?

In the previous breakout sessions important new tools to move clinical research towards clinical trials in translational and personalised medicine were presented. The overall impression is that the process of simple data collection has become insufficient for clinical research and study feasibility and patient recruitment has to be supported; EHR data, mobile eHealth data, biobank data and data warehousing solutions have all to complement the data collection process. Töresin Karakoyun pointed out that for clinical research and data collection the ability for cooperation has become of decisive importance. Thus cooperation between EU projects and between research infrastructures should be increased. While projects and infrastructures are often aware of the existence of tools developed elsewhere, improved cooperation capabilities should enable the exchange of resources and people as carriers of expertise between projects and infrastructures. In this way, researchers will be enabled to find the best solution and the best tool for their research. Users of tools and tool developers should learn more efficiently from each other. The ESFRI infrastructures may work better together through BioMedBridges. As a first step, BioMedBridges will create a repository of all tools, documents, sources of the member research infrastructures.

The validation and certification as a necessary condition for sustainability is far from easy requiring the creation of a large amount of documents for system validation, like requirements specifications, functional specifications, and user specifications. This leads to the issue that in the academic world the process of software development is not as clean and precise as in industry. For example, tool development is often an integrative part of a research project, as a result of which requirements become a moving target. Thus, often no frozen version of a tool is available for its validation. Once one has decided to freeze tool development for the validation / certification, research still goes on resulting in new requirements and changes in tool development. Norbert Graf clarified the need for certification of the tools developed in academia. The ability for certification depends also on the way projects and their developed tools are funded. As a consequence, the EU should provide funds to help projects to move into sustainability. But to provide tools as Open Source is not an easy solution either (Christian Ohmann); because Open Source, too, needs support, services, and regular updates, requiring somebody who pays for the sustainability of Open Source. We are now at a point, where the gap between pure research focusing on new problems and publications and business focusing on the generation of money has to be bridged for the EU projects. Projects must now be helped to move from a research approach to a product development approach.

Jacques Demotes turned the discussion to the user communities for the developed tools. IT experts are concerned that tool development is not driven by the user communities. For example, clinical trials representatives have specific needs but cannot develop tools by themselves. Thus, as a first step priorities and strategies should be defined for the clinical trials communities. Although user requirements for clinical data management systems (CDMS) exist in ECRIN, they cover only the basic aspects of data management. But CDMS requirements should consider new developments in clinical trials, like the involvement of imaging, biobanking and genome data. Here new tools are necessary and must be integrated in existing clinical data management processes. In addition, tools that are employed for clinical trials must be system validated for GCP compliance, requiring specifications for the tools that cover these new fields of use. As a consequence, ECRIN standard requirements that have been developed for the certification of ECRIN data centres should be extended accordingly. In addition, the patient point of view must be considered for clinical trials more strongly; patients will tell us more and will give us more data. On the other hand, patients should be able to decide how their data and biosamples should be accessed and used, and how the right informed consent is employed.

Conference participants recognised a competitive situation between the tools shown; for example, the tools ObTiMA, OpenClinica, VISTA support data management in clinical trials, whereas tranSMART and MOLGENIS are data warehouse based solutions. It was suggested to increase the transparency of developments and improve the exchange of tools, information, as well as source code created in different projects. Norbert Graf recommended building tools in a modular fashion, like it is done in p-medicine, and with standardized interfaces. Ontologies should be able to link directly to items. In this way, researchers will be able to select and assemble appropriate modules of different tools for their research question. Often not everybody needs all functionality available. This idea found support in the conference. Tools may come and go, but the focus of software development should be on standardized interfaces and data standards. One important standard in this context is IHE (Integrating the Healthcare Enterprise), providing specific profiles for data retrieval. Standards are able to bring different tools together, like in the case of CDISC ODM, a data exchange standard that can be used to integrate different CDMS to be used in single clinical trial [9]. Töresin Karakoyun pointed out that the area of processes should not be forgotten, because processes are often still far from being clearly defined, like it is the case of the workflow for personalised clinical trials.

## ECRIN and data in multinational clinical trials

**Jacques Demotes** (ECRIN) was looking forward to identifying the challenges that are encountered by clinical trials infrastructures. For clinical trials addressing innovative health products, exploration of new indications for existing drugs, comparative assessments of efficiency and safety of approved healthcare strategies, new infrastructure solutions are necessary that need collaboration between industry-funded and non-industry funded approaches. It is the lack of funding and the missing infrastructures that are the reason why only 3% of non-industry funded trials are international. ECRIN-ERIC is a combination of national networks of CTUs with national organizational points by EUCos (ECRIN European Correspondents). Employing this infrastructure, ECRIN provides all kind of services, like assessment of feasibility, logistic, contract development, coordination with ethics committees, site monitoring, etc. IT should enable researchers to better use health data for clinical trials, avoiding any duplication in data collection. But only a small number of patients is actually being enrolled to participate in clinical trials or to form part of a cohort study. The systematic use of health data for research, including the integration of registries, cohorts, and clinical trials nested in cohorts, international cooperation in non-commercial clinical trials must be facilitated by establishing national / regional / global networks of co-operation in clinical science accompanied by increased patient involvement in the clinical trial process. To support these objectives, ECRIN expects that IT should improve the access to clinical data (supporting data transparency and making patient-level data available for meta-analysis), enable up-to-data analysis of all different types of clinical data (the ability of meta-analysis should be mandatory), provide suitable anonymised patient data for analysis (for example, retrieval of aggregated data sets, support of interoperability by using standardized outcome measures, and by improving the integration of CRF data with high throughput patient data in clinical trials (genomics, proteomics data).

In the subsequent discussion growing concerns in the research infrastructure community were addressed that have to do with the fact that research conduct is changing from research done by small teams of post docs to large anonymous teams in big projects. As a consequence, collaboration, contribution and the authoring rules have become complex and often unclear; research contribution cannot be based solely on authorship anymore.

A first step to increase transparency in clinical research is to make the data collected by CRFs public, not only the summaries of results and eligibility criteria. But this must be followed by providing access to the study protocol, and additional data like trial performance data. Access to all clinical trials data requires sophisticated access policies, to prevent any misuse of data (e.g. the prevention to identify research participants), as well as data provenance and a complete audit trail.

## Session 2: translational research projects and their tools for clinical research

**John Overington (EU Openscreen)** presented the EU Openscreen infrastructure that combines chemistry with biology and consists of national content collection centres. EU Openscreen is a distributed infrastructure enabling trans-national access for the development of bioactive small molecules. All databases are open access; and additional screening centres with high-end equipment exist. Recently, an ERIC for EU Openscreen is being negotiated.

EMIF was introduced by **Alvaro Cortes (EMIF)**. EMIF is an IMI project with the aim to develop a common information framework of patient-level data to facilitate access to diverse medical and research data sources. It started in January 2013 with 48 partners. Because it provides access to individual data sources and tries to enhance already existing data sources, EMIF can be seen as a federation of databases; whereby each data source retains full control over its data. Database owners are supported by the provision of means for anonymisation / pseudonymisation, tools for linking ontologies and by support of semantic interoperability.

**Anka Bucur (Phillips)** introduced the INTEGRATE project dealing with integrative cancer research with the aim of facilitating clinical research to improve the treatment of cancer patients. INTEGRATE focuses on using knowledge sharing for patient recruitment. Recent use cases are in breast cancer, but the solution developed will be applicable to all disease domains by providing a semantic interoperability platform. Tools for patient screening and pathology review were shown in the hands-on sessions during this workshop. An additional tool deals with cohort selection by providing means for filtering data, querying databases, and visualization of selected cohorts.

**Jan-Willem Boiten (TraIT)** presented issues of translational research. The TraIT project develops an IT infrastructure for translational research with the aim to facilitate the collection, storage, analysis, archiving, and sharing of data by providing means to connect clinical phenotype data with disease biology data. It started in 2011 as a Dutch national initiative between CTMM, the Dutch Cancer Society, the Dutch Heart Foundation, the Netherlands Bioinformatics Centre (NBIC), the Parelsnoer Institute (PSI) and others and developed in the meantime to a consortium that consists now of 27 partners. The project uses already available tools that are integrated into an IT infrastructure. TraIT will demonstrate the tools OpenClinica, XNAT, BBMRI catalogue and TranSMART in hands-on sessions during this workshop. Already 80 studies with about 700 users are conducted with OpenClinica as data management system for clinical trials.

**Peter Wittenburg (EUDAT and RDA)** introduced EUDAT, a project that provides common data services for the entire research community. EUDAT is a collaborative data infrastructure consisting of a federation of trusted centres with the focus that its community people are driving the project. The services available are B2FIND for finding research data, B2SAFE for safely replicating research data, B2SHARE for storing and sharing research data with added semantics and B2STAGE for preparing data for high performance computing. An important aspect of EUDAT is the Persistent Identifier (PID) service. In principle, every bit of data may get a PID as a kind of tag when it enters EUDAT. Another tool is the AAI service offering secure access to scientific systems. Recently, EUDAT collaboration projects have started, with about 30 submissions for participation. ECRIN plans the testing of policy mechanisms for sensitive data together with EUDAT. Because big national centres are the actors in EUDAT (e.g. CSC, SARA, RZG) the sustainability of services is ensured by agreements.

**Niklas Blomberg (EBI)** spoke about ELIXIR. ELIXIR will provide necessary facilities for researchers providing a multitude of tools required by academics from bench biologists to chemo-informaticians. In this way, it supports work with the rapidly growing amount of information about living systems. The ELIXIR infrastructure includes databases (e.g. ENSEMBL), reference data, tools for data access, tools for the exchange and analysis of sensitive data, standards for data exchange, encoding and integration of data as well as training for researchers. To maintain core data resources, sustainability plays a role in the pilots of ELIXIR. The EU genotype / phenotype archive, which contains individual genomes, has raised the problem of identifiability of individuals from their genetic information. As a member of the BioMedBridges project, ELIXIR helps with a tool for the consistent identification of biosamples (BioSD) that allows finding and linking 2.8 Mio samples in 32 sources worldwide.

## IT challenges for innovative clinical trials

The keynote speech was held by **Norbert Graf** **(USAAR)** about the topic of IT and innovation in clinical trials. In areas outside clinical trials, IT development has proceeded to create eBooks and eBanking. In the clinical trials area such a development is still missing. But as an example what is already possible, paediatric oncology can be seen as a success story, where nearly 100% of patients are able to participate in a clinical trial and where treatments show striking successes. On the other hand, still only 5% of adults are enrolled in prospective clinical trials. In addition, in paediatric oncology, personalized medicine has become very successful. But personalized medicine is rather complex requiring many different kinds of data, even data from system biology models. The important point is that all necessary patient data is available for the physician in due time to be used for clinical decision support. Thus, tool development in personalized medicine must be clinically driven, evaluated, validated and in a standardised form to allow the reuse of data. In addition, personal data must be secured by a security framework (that once being developed can be re-used for other projects). The use of common ontologies and semantic mechanisms allow data sharing and integration and may be shared between projects, too.

The principal investigator may be supported by tools offering electronic informed consents. But it has to be considered that often a global informed consent is not possible, for example in research with biomarkers. In this case, the investigator has to re-contact the patient to receive a new right to use biosamples and data for future research. This situation illustrates the apparent value conflict that exists between the individual treatment (privacy protection) and the collective interest (access to data and research with data). Here a balance has to be found between both exclusive interests without compromising the transparency for the patient. In this respect, recent approaches including the ones of PatientsLikeMe, where patients can upload their clinical data, but where the personal clinical data belong to the service provider, who is allowed to sell them for example to insurance companies, should be avoided. This is not the form of transparency that will support and improve research.

What is often forgotten is that innovative clinical trials demand not only novel IT support, but a higher degree of teamwork between different stakeholders, like infrastructure providers, legal/ethical people, tool users, trainers, validation experts, and patients as one additional partner. Therefore, to concentrate research only on data-driven approaches will not improve research, but will result in researchers being lost in data space.

Returning to the p-medicine project, data management is done with ObTiMA, a tool that has been demonstrated in this workshop. The experience of the project with software development is that in general, tools should be built in modules, to allow combination of different modules to adapt to changing requirements, and to ease interoperability between tools. The integration challenge for personalised medicine data consists in the necessity that patient data from different hospital information systems (HIS) and different push/pull specifications must be considered and joined. Here, integration can be simplified by putting HIS data into a standardised data warehouse. While in the research area it is often possible to use different tools that all are able to access data of patient cohorts or anonymised medical data, in the area of decision support only the same tool should be used to ensure uniformity in decision support. Decision support tools must also be able to access personal data of a single patient under conditions of real time constraints. This gap between basic research and clinical application can only be closed by interdisciplinary teams.

## Break out session 3

In the third demonstration session, tools for clinical research support (Clinical Trial Information Mediator, Pathology Reviewer, PASTEL) and EHR associated tools for clinical data (Query Workbench, Patient recruitment service) were presented and discussed.

## Final joint discussion: conclusions and how can we achieve sustainability of tools and services?

The first CRI Solutions Day provided an opportunity to hear from a diverse group of professionals working in research infrastructure management, clinical trials and biomedical research about how they cope with the challenges of new developments in the field. The format of the workshop consisting of lectures and hands-on sessions of tools with developers was very positively received; it was appreciated that the focus was on the developed tools and it was suggested to conduct such a workshop regularly each year.

The first impression was that EU projects seem to cope with many similar problems and have come up with similar solutions. For example, all domains struggle with the same rigid conditions for data protection and the challenge of semantic interoperability. Christian Ohmann indicated that several tools address the same problem, for example four projects developed a query generator. But, do researchers need this kind of competition? If in such cases a generic problem exists, it might be better to bring projects together, work jointly on software development and increase transparency. Project funding too, should consider this aspect in EU projects. Peter Wittenburg suggested that researchers in different domains may be able to use the same tools for their research. In principle, this problem is also a matter of the funding organisations and the way projects are supported. On the other hand competition between projects and the development of similar solutions may also be a good feature. Norbert Graf argued that it should be considered that tools developed in EU projects often grow out of special research questions and are based on domain specific requirements. Therefore, project members should compare and examine what things that can be done together and what can be shared (e.g. common ontologies used in different disease settings). Ann Martin agreed about differences in the fundaments of EU projects and was surprised how different the projects actually are. Thus, the observed similarity of tools may be only a resemblance on the surface, but may hide deeper underlying differences in approaches and requirements. Töresin Karakoyun took up this remark to address the problem of sustainability of tools developed in EU projects suggesting that the next CRI Solutions Day should be about how to sustain tools and provide support after the end of a project. It was suggested that industry should be more involved, perhaps even connections with venture capital may be helpful to sustain developments. But the main problem faced by all research projects that develop tools is the need to shift tool development from the research area to a setting of professional service provision. Jacques Demotes stated that sustainability should be an outcome of a project. Limited ERIC funds are available to promote a couple of tools that could be part of a clinical trials tool repository. But in the end, the provision of tools must generate some revenue to invest in maintenance and further developments. The infrastructures are learning to deal with the different challenges; sustainability is one of these challenges and the many query tools may be part of the game (Peter Wittenburg). Thus, projects should create and improve their business models, work together with stakeholders and funders and tackle the need for seed money. Though, as was suggested, infrastructures and projects may share components and tools; it may need 2-3 years for a project to settle a joint understanding to be able to share.

It followed a discussion of what the business models of the different tools presented in the break-out sessions were. Several of the tools provide access to patient data and thus, supply pharma industry demands. Industry laments the missing access to patient data; negotiations with hospitals about patient data often last years. The problem with hospital data and patient data in general is the restriction of regulations and rules on the use of sensitive data for research purposes. In the area of patient care and hospitals, trust in the industry does not exist; and the main concern of data protection officers at hospitals is how any misuse of data can be absolutely prevented (Christian Ohmann); an aspect generally overlooked by industry. To generate this necessary trust, several aspects must become transparent: the reasons for access to patient data, who is doing what with the data, and who is responsible for the data. In the US, insurance companies are already selling patient data to defined questions from industry. In addition, as a consequence of regulations and rules to protect data privacy the developed tools must incorporate means for audit trails and provenance control. In this context, the TRANSFoRm project has developed a comprehensive framework to monitor provenance [10]. Nonetheless, to perform effective health research, systems in hospitals must open. Patients too are waiting for treatment and are interested in new research findings and therefore to a large degree support research with their data. In such a situation a robust legal framework is needed to ensure that privacy is protected and that non-anonymised data as well as genetic data is not shared in an unrestricted way. In fact, laws that forbid physicians to share such patient data already exist. An extension of the problem of data confidentiality is that tool development in EU projects should not omit the patient empowerment perspective (Norbert Graf). In the end data belong to the patient, and often the patient wants not only to share data but also wants access to data. Thus, both aspects together, transparency and trust, are of utmost importance for the development of tools. Perhaps one should show these problems to policy makers, to find a way how to overcome them. Ann Martin pointed out that in fact policy makers have taken up this topic and are willing to protect patients; IMI is working on guidelines about secondary use of treatment data for research.

The final comment, the take home lesson, was stated by Töresin Karakoyun: 11 EU / IMI projects, 7 research infrastructures, 22 tools and 92 participants came together and possibilities for collaboration turned out to be a focal point for all projects. Collaboration between research infrastructures and projects can proceed via a 7-stage model moving from simple coexistence and communication to more intensive cooperation, coordination and finally to coadunation [11]. At the moment EU projects are at the point of cooperation and coalition building, but have not yet reached the possibilities of true collaboration. To support active collaboration a kind of platform is needed, to enable collaboration by providing help to decide how to organize it, how to pay for it, how to support and maintain it. There should be rules and guides and a budget for collaboration; technology alone is not enough.

## Conclusions

The first CRI Solutions Day provided a unique opportunity to see, discuss and evaluate the new tools that soon will change the way research is done, demonstrated by the leading research infrastructures and EU projects in the field of biomedical research. It became clear that still many challenges exists, especially the sharing and joining of tools, collaboration and sustainability issues. Nonetheless, using new tools that will provide advanced IT support, biomedical research and clinical trials will become more interesting in future.

## Financial & competing interests disclosure

The authors have no relevant affiliations or financial involvement with any organization or entity with a financial interest in or financial conflict with the subject matter or materials discussed in the manuscript. This includes employment, consultancies, honoraria, stock ownership or options, expert testimony, grants or patents received or pending, or royalties.

## Author’s contributions

WK is the main author and collected the abstracts; WK, TK, SG and CO contributed to the text and corrected the text.

## Abbreviations

AAI: Authentication and Authorization Infrastructure; BBMRI: Biobanking and Biomolecular Resources Research Infrastructure; BRIDG: Biomedical Research Integrated Domain Group; CDIM: Clinical Data Integration Model (TRANSFoRm project); CDISC: Clinical Data Interchange Standards Consortium; CRI: Clinical Research Informatics; CSC: IT Center for Science; CT: Clinical Trial; CTIM: Clinical Trial Information Model; CTMM: Center for Translational Molecular Medicine; CTU: Clinical Trial Unit; eCRF: electronic Case report Form; ECRIN: European Clinical Research Infrastructures Network; ECRIN-IA: European Clinical Research Infrastructures Network-Integrating Activity; EHR/EMR: Electronic Health Record / Electronic Medical Record; EHR4CR: Electronic Health Records for Clinical Research; EORTC: European Organisation for Research and Treatment of Cancer); ESFRI: European Strategy Forum on Research Infrastructures; ETL: Extract, Transform, Load - process; EUCos: ECRIN European Correspondents; EUDAT: European Collaborative Data Infrastructure; FP7: EU Framework Program 7; GCP: Good Clinical Practice; HL7: Health Level 7; i2b2: Informatics for Integrating Biology and the Bedside; IHE: Integrating the Healthcare Enterprise; IMI: Innovative Medicines Initiative; INTEGRATE: Integrative Cancer Research; IT: Information Technology; KKS: Coordination Centre for Clinical Trials; LAT: Legal Assessment Tool; LexEVS: EVS terminology server; ObTiMA: Ontology Based Trial Management for ACGT; ODM: Operational data Model; p-BioSPRE: p-medicine Biospecimen Search and Project Request Engine; PCROM: Primary Care Research Object Model; PID: Persistant Indentifier; REST: REpresentational State Transfer; RZG: Rechenzentrum Garching; SARA: Stichting Academisch Rekencentrum Amsterdam; STaRC: Study, Trial and Research Centre; TraIT: Translational research IT; TRANSFoRm: Translational Research and Patient Safety in Europe; USAAR: University of the Saarland; XNAT: Extensible Neuroimaging Archive Toolkit

## Declarations

The workshop was financed by the Strategischer Forschungsfond of the Heinrich-Heine University, Düsseldorf (F-2014/626-9), and financially supported by contributions from IMI, TRANSFoRm, and p-medicine.

The Publication was financed by the Strategischer Forschungsfond of the Heinrich-Heine University, Düsseldorf (F-2014/626-9), and partly financially supported by CTMM-TraIT Project, Sustainable IT Infrastructure for Translational Biomedical Research; TRANSFoRm (EU FP7, grant agreement no 247787), p-medicine (EU FP 7 grant agreement No 270089) and BioMedBridges (EU FP7 grant agreement number 284209).

## References

[B1] Gartner, Inc. IT Glossary 2013 Advanced Clinical Research Information Systems (ACRIS) http://www.gartner.com/it-glossary/advanced-clinical-research-information-systems-acris

[B2] European Commission Medical research in the European Union, Health Directorate, Medical Research Unit 2014 http://ec.europa.eu/research/health/medical-research/index_en.html

[B3] Demotes J ECRIN an infrastructure to support multinational clinical research ESFRI-BMS and JPI 2011 congress Brussels http://ec.europa.eu/research/infrastructures/pdf/6._ecrin.pdf

[B4] TRANSFoRm project http://www.transformproject.eu

[B5] Rossi S Christ-Neumann ML Rueping S p-Medicine: From data sharing and integration via VPH models to personalized medicine Ecancermedicalscience 2011 5 218 223 22276060 10.3332/ecancer.2011.218 PMC3223941

[B6] EHR4CR project http://www.ehr4cr.eu

[B7] Kuchinke W Ohmann C Yang Q Salas N Lauritsen J Gueyffier F Heterogeneity prevails: the state of clinical trial data management in Europe - results of a survey of ECRIN centres Trials 2010 11 79 89 10.1186/1745-6215-11-79 20663165 PMC2918594

[B8] Ethier JF Dameron O Curcin V McGilchrist MM Verheij RA Arvanitis TN Taweel A Delaney BC Burgun A. A unified structural / terminological interoperability framework based on LexEVS: application to TRANSFoRm J. Am. Med. Inform. Assoc 2013 20 5 986 994 10.1136/amiajnl-2012-001312 23571850 PMC3756256

[B9] Kuchinke W Wiegelmann S Verplancke P Ohmann C Extended cooperation in clinical studies through exchange of CDISC metadata between different study software solutions Methods Inf Med 2006 45 4 441 446 16964363

[B10] Curcin V Danger R Kuchinke W Miles S Taweel A Ohmann C Liu, Q.; Bai, Q.; Giugni, S.; Williamson, D.; Taylor, J. Provenance Model for Randomized Clinical Trials Data Provenance and Data Management for eScience. Studies in Computational Intelligence 2012 426 Springer Berlin Heidelberg 3 33

[B11] Karakoyun T Kuchinke W Ohmann C Auer HJ Paul Cunningham and Miriam Cunningham A Scenario-Driven Approach to Enable Collaboration among Research Infrastructures and Initiatives eChallenges e-2012 Conference Proceedings 2012 IIMC International Information Management Corporation Ltd

